# Supplementary material for: Negotiating climate change: Science, policy, and the invisible power embedded in public discourse in Chinese social media
Source: PLoS One. 2026 May 14;21(5):e0348708. doi: 10.1371/journal.pone.0348708 (PMC13175376; doi:10.1371/journal.pone.0348708)
Supplement: Attachment — Submitted filename: 3.Response to Reviewers.docx [file pone.0348708.s001.docx]

**Response to the Editor and Reviewers**

We sincerely thank the editor and reviewers for their constructive comments. All suggestions have been carefully considered and addressed in the revised manuscript. Detailed responses are provided below.

**Editor Comments:**
1. Please ensure that your manuscript meets PLOS ONE’s style requirements, including those for file naming.

Response:

Thank you for this helpful comment. We have reviewed the manuscript to ensure it fully complies with PLOS ONE’s style requirements, including formatting conventions and file-naming guidelines. All files have been renamed and formatted accordingly.

2. In your Methods section, please include additional information about your dataset and ensure that you have included a statement specifying whether the collection and analysis method complied with the terms and conditions for the source of the data.

Response:

Thank you for this helpful suggestion. We have also added a statement clarifying that the collection and analysis of the data complied with the terms and conditions of the source platform (pp. 10).

3. Please note that PLOS One has specific guidelines on code sharing for submissions in which author-generated code underpins the findings in the manuscript. In these cases, we expect all author-generated code to be made available without restrictions upon publication of the work.

Response: Thank you for this important reminder. We have made the code and data publicly available in accordance with the journal’s requirements. The repository link is provided in the manuscript (see Data Availability Statement, pp.1), and the raw data and code will be accessible without restriction upon publication.

4. We note that one or more of the authors are employed by a commercial company…

Response:

Thank you for the reminder. We have revised the Funding Statement and Competing Interests Statement in accordance with PLOS ONE’s requirements. The Funding Statement now specifies that the commercial affiliation provided salary support only and had no role in the study. The Competing Interests Statement has been updated to declare employment and confirm adherence to PLOS ONE policies on data and material sharing. Both statements are included in this response letter as requested.

5. Additional Editor Comments: In particular, I recommend addressing the suggestions to (a) restructure the discussion around the Research Questions, (b) addressing the social representations literature more directly, and (c) noting the pertinence of different communication genres like social media.

Response:

Thank you for synthesizing these important revision priorities. We have carefully considered these points and addressed them as outlined below.

(a) Following this recommendation, the Results sections have been restructured to align explicitly with the Research Questions (pp. 9). The revised Results are now organized into two main subsections: “5.1 The Central Structure of Social Representations of Climate Change...” (pp.11-14), “5.2 Temporal Variation in Climate Change Representations…” (pp.14-19), which together address the structural and temporal dimensions of the research questions. In addition, the Discussion section has been reorganized to explicitly revisit each research question, clarifying how the empirical findings respond to them and how they inform the broader theoretical framework (pp.19-26).

(b) We agree that a clearer, more systematic integration of Social Representations Theory into the Discussion would enhance the manuscript’s theoretical depth. The Discussion (pp. 19-26) has been revised to engage more directly with Social Representations Theory. We now clarify how the identified semantic structures correspond to core and peripheral elements, hegemonic configurations, and processes of stabilization and reorganization over time.

(c) As you and the reviewers rightly pointed out, we recognize the analytical value of distinguishing between different types of actors operating on social media. However, due to the anonymity of platform registration and the design of our corpus-based approach, actor-level differentiation was not systematically operationalized in the present study. Our analysis, therefore, concentrated on the structural organization of representations rather than actor-specific dynamics. We have now clarified this limitation in the revised manuscript (pp. 26) and explicitly identified the examination of heterogeneous actors and their differential discursive influence as an important direction for future research.

**Reviewer 1 comments：**
1. The main limitation, which I would describe as structural and of which the author(s) seem quite aware (see Conclusions), is the basic assumption that all discourse on social media can be interpreted as public discourse. In addition to the issues already acknowledged by the author(s), there is the question of not considering, or consider little, social media – even attempting to control it from a technical or methodological point of view – as an arena in which highly heterogeneous actors operate (and therefore with different opportunities and structures of symbolic and discursive power). The focus solely on the linguistic and semantic dimensions of corpora tends to flatten the issue of who produces discourse. Clearly, policy discourse reflects (as well as directly emanating from) government agencies, organised actors, NGOs, politics, but also fragmented actors: beyond the political and institutional characteristics of the Chinese context, in its specificity, discourse on social media is also developed by individual experts, influencers, celebrities, ordinary people as individuals, etc. I understand that this is not an easily solvable problem and that, above all, even if this focus could be added (to research already conducted), it is not certain that this would be efficient or capable of producing interesting inferences, but some form of identification and coding could have been included in the approach, as a variable (in addition to the period), of the actors who produce the discourse, even if only as a simple coding (type of actor, individual vs. collective, institution, etc.). Or if this has been done, it is unclear (and has not been explored analytically).
**Response:**

We sincerely thank the reviewer for raising this important structural concern. We agree that social media discourse is produced by heterogeneous actors with unequal symbolic and discursive power, and that focusing solely on linguistic and semantic structures may underrepresent the question of “who” produces discourse. In this study, we did not code actor types. One practical reason is that Zhihu operates largely through anonymized registration, and user identities are not institutionally verified, making systematic classification difficult. Moreover, manual coding of actor types could introduce substantial classification errors. Our analysis, therefore, concentrated on the structural organization of representations rather than actor-specific dynamics.

We have now explicitly acknowledged this limitation in the revised manuscript. We agree that incorporating actor-type coding would be a valuable direction for future research and hope to implement this extension in subsequent studies. (see the Conclusion section, pp. 26).

2. I recommend reviewing certain parts of the discussion, where the connection with some of the key themes in the literature may appear somewhat forced and could be better articulated.
For some statements, which are probably plausible and in principle not falsifiable, or correct, the placement in the discussion of the results does not seem to be adequately supported – at least explicitly – by the data. Where, for example, is the data supporting this part? (p. 23):
For instance, a misalignment is often observed in public discussions about the attribution of responsibility, with the public tends to blame corporations for causing global warming while holding governments accountable for solving the problem; meanwhile, governments increasingly place the burden of action on corporate actors. This flow of responsibility discourse reflects an ongoing struggle over the fundamental question of “who is responsible.”
**Response:**

We thank the reviewer for this careful reading. We agree that the original wording concerning the dynamics of responsibility discourse may have appeared overly generalized. In the revised manuscript, we have reformulated this section to limit our interpretation to patterns directly observable in the co-occurrence structures.

page 23: *“A similar pattern can be observed in Chinese data… In the co-occurrence networks and correspondence maps, enterprise-related terms frequently cluster with carbon emissions, energy governance, environmental regulation, and responsibility. Public discussions often cite IPCC reports, temperature thresholds, and carbon intensity indicators, linking emissions to economic growth. Within this framework, corporations are positioned as key actors in climate mitigation. Climate change is therefore understood less in terms of individual behavior and more as an issue of industrial transformation and policy-led governance. Framed within resource management and economic regulation, it becomes detached from moral obligation.”*

More generally, we have carefully reviewed the discussion to ensure that theoretical connections are grounded in the presented findings. These revisions include clarifying the empirical basis of key claims, removing or moderating statements that extended beyond the scope of the results, and improving the articulation of theoretical links to ensure coherence with the presented analyses. (see the Discussion section, subsection 6.2 and 6.3, pp. 21-25).

3. Some statements are not supported by references (bibliographical or primary data), at least not explicitly. Please correct. E.g. p. 24
For example, some residents in northern Chinese cities tend to…
**Response:**

We thank the reviewer for this careful observation. Following this comment, we have carefully revised the discussion section. Specifically, we have removed interpretations that were not sufficiently grounded in the presented results, merged repetitive expressions, and streamlined the overall argumentation to improve clarity and coherence (see the Discussion section).

In addition, we rechecked the references and their positions throughout the manuscript to ensure they were cited correctly.

4. p. 12 When mentioning the software used, I think it is always good practice to mention the version used: SPSS, version?

**Response:**

Thank you for this helpful suggestion. We have now specified the SPSS version used in the revised manuscript (see the Data analysis section, pp. 11).

page 11: *“… Subsequently, the node and edge data are imported into Gephi (version 0.10.1) to construct the word co-occurrence network and analyze the network’s structural features and semantic connections among keywords… we used SPSS (version 29) and Python to perform the corresponding analysis and its visualization.”*

5. p. 17 Check the sentence: the sentence The terms “science,” “developed countries,” and “developing countries.” seems incomplete, is there a verb missing? Or, directly, without a full stop, "... suggest that…"
**Response:**

Thank you for pointing this out. We have deleted the incomplete sentence (pp.17) and conducted a thorough review of the manuscript to ensure grammatical accuracy and structural clarity throughout.

6. Attention, check typos or error. I have noted some here, but it means that careful reading is required to ensure that there are no others.
p. 10 Correspondence analysis (not Correspondent)
p. 10 Chinese (not Chiese)

**Response:**

We sincerely apologize for these typographical errors and thank the reviewer for pointing them out. The mentioned mistakes have been corrected, and we have carefully proofread the entire manuscript to eliminate any remaining typos and ensure overall linguistic accuracy.

**Reviewer 2 comments：**
1. The abstract mentions action-oriented and governance-focused perspectives, but these are not present in the text. The authors are advised to show how these are relevant to the study or its findings.

**Response:**

We thank the reviewer for this constructive comment. Using more precise wording and articulating the connections between our findings and the broader discussion more clearly would have been beneficial. To address this problem, we revised the Discussion and Conclusion sections to more explicitly demonstrate how these perspectives emerge from our findings. For example, *“Taken together, the semantic networks across these three periods reveal a relatively stable scientific context, with these terms consistently anchoring discussions of the scientific consensus on climate change. At the same time, associations related to governance, responsibility, and solutions have grown increasingly prominent” (PP.14).*

*“…The social representations of climate change have moved from an early cognitive framework centered on scientific mechanisms toward a greater emphasis on economic practices and action pathways.” (PP.19).*

*“In this process, policy does not function as a static, one-way transmission of directives, but rather as an ongoing social negotiation. It is reflected both in the implementation of policies that contribute to the reconstruction and renewal of climate representations and in the provision of cognitive and normative foundations that legitimize particular representations. In other words, policy not only responds to public risk perceptions but also offers new anchoring points for understanding climate change, thereby enabling context-specific updates through the expansion of the peripheral structure. Consequently, communication practices surrounding these topics might thus need to adopt a more holistic perspective and language to connect to the public’s representations of these issues.” (pp.22)*

We have also refined the abstract to better align with the revised discussion and enhance overall coherence (see the Abstract section, pp. 2).

2. The literature review is sound, and culminates in three research questions. However, in presenting the results, the findings are not structured around these questions. Instead of sticking to analytical categories on their own (e.g., as relating to word count, correspondence analysis, etc.), the Results section should be structured around the research questions (whilst also bringing out the findings of the separate analyses). This would ensure that the writing directly addresses the RQs.
**Response:**

We thank the reviewer for this valuable suggestion. Following this recommendation, we have thoroughly restructured the Results section to align more explicitly with the research questions. The revised Results are now organized into two main subsections:

Page 11-14: *“5.1 The Central Structure of Social Representations of Climate Change...”*, which addresses RQ1 and RQ2 by presenting the core semantic structures and linguistic associations identified through semantic network analysis and correspondence analysis.

Page 14-19: *“5.2 Temporal Variation in Climate Change Representations…”*, which addresses RQ3 by examining the evolution of social representations across the three time periods.

Within each subsection, the findings from the respective analytical techniques are integrated to directly respond to the research questions. In addition, the second subsection further elaborates on the semantic configuration of the core representations, thereby providing supplementary insights relevant to RQ1(pp. 16). This integrated structure ensures that the findings are clearly organized around the research questions while coherently incorporating the results of the different analytical methods.

3. The Discussion lacks theoretical depth. Social representations feature in the literature review, but not in the Discussion (only occasionally, when discussing hegemonic representations). It would be ideal if social representations feature (1) theoretically, and (2) in view of the research questions on Page 10: How were these answered? What contributions do the findings make? Which theoretical framework is supported by the findings, or opposed? etc.
**Response:**

We thank the reviewer for this important comment. We agree that a clearer, more systematic integration of Social Representations Theory into the Discussion would enhance the manuscript’s theoretical depth. According to the reviewer’s suggestion, we have substantially revised the Discussion section to explicitly engage with Social Representations Theory in relation to the research questions. The revised discussion now clarifies how the findings address each research question and demonstrates how the empirical results support the structural approach to social representations, particularly the distinction between central and peripheral systems and the emergence of hegemonic representations in the Chinese context.

Furthermore, we have strengthened the theoretical contribution of the study by highlighting how the observed stability of scientific representations and the dynamic evolution of economy-oriented representations reflect the dialogic and context-dependent nature of social representations. These revisions ensure that Social Representations Theory is consistently integrated into both the interpretation of the findings and the articulation of the study’s theoretical implications (see the Discussion section, pp. 19-25).

4. The research questions (page 10) are "What social representations of climate change are most salient in public discourse on social media?", "To what extent is public consensus on climate science influenced by major government initiatives or significant events?", and "How does media discourse on climate change interact with the social construction of climate risk within social media environments?" However, keywords shift from representations to discourse to scientific narratives, meaning that the paper is less coherent than it could be. Authors are advised to re-write parts of the paper (where relevant) to ensure alignment between approaches (e.g., predominantly social representations, or predominantly a discourse-based approach).
**Response:**

Thank you for pointing out this important issue. We agree that the initial version of the manuscript occasionally employed overlapping terms such as *social representations*, *discourse*, and *scientific narratives*, which may have reduced conceptual coherence.

In the revised manuscript, we have carefully reviewed and standardized the terminology to ensure alignment between the theoretical framework, research questions, and analytical approach. Social Representations Theory now serves as the central conceptual lens throughout the paper. The identification of social representations is grounded in the semantic structures revealed through semantic network analysis and correspondence analysis, which enable us to capture the organization, stability, and evolution of shared meanings in social media discourse. In this context, terms such as *discourse* and *scientific narratives* are used only when referring to the linguistic expressions through which social representations are articulated, rather than as alternative analytical frameworks. To further enhance conceptual consistency, we have revised the research questions. The revised research questions are as follows:

Page 9*: “RQ1: What constitutes the core social representations of climate change on social media? RQ2: How do linguistic and semantic connections in social media representations influence the public’s understanding of climate risks? RQ3: How do major national policies or significant events influence social representations of climate change?”*

We have also revised the literature review (pp. 8), results (pp. 14-15; pp. 19), and discussion sections (pp.19-25) accordingly to ensure conceptual consistency and theoretical clarity. These revisions strengthen the coherence of the manuscript and clarify its primary theoretical orientation as a study of climate change social representations in digital communication environments.

5. Contributions of this work, practical implications, and directions for future research, need to be highlighted more (in Conclusion chapter)

**Response:**

We sincerely appreciate this observation. We have substantially revised the Conclusion section to more clearly highlight the theoretical contributions, practical implications, and directions for future research.

Specifically, we have clarified the study’s main findings in relation to Social Representations Theory. We think our study extends the application of social representations theory within digital media contexts. It demonstrates that climate change is interpreted through social lenses rather than treated as a purely scientific object. Instead, it is continuously objectified and reorganized within institutional environments and social interactions. We have also expanded the practical implications by discussing how the findings may inform climate communication strategies and policy discourse in the Chinese context. We have also expanded the practical implications by clarifying how the findings deepen our understanding of Chinese public perceptions of climate change.

 In addition, the section on future research has been refined to acknowledge the limitations of the current study and to suggest potential extensions, such as incorporating actor-based analysis and exploring representations across different platforms or cultural contexts. Furthermore, the future research section has been refined. We now suggest adopting broader analytical frameworks beyond the scientific label “climate change” to better capture the complexity of common-sense representations, examining the role of different network actors in shaping social representations, and employing experimental approaches to investigate how distinct representational structures influence public intentions to act (see Conclusion section, pp. 25-26).
